# Phonon-exciton Interactions in WSe_2_ under a quantizing magnetic field

**DOI:** 10.1038/s41467-020-16934-x

**Published:** 2020-06-19

**Authors:** Zhipeng Li, Tianmeng Wang, Shengnan Miao, Yunmei Li, Zhenguang Lu, Chenhao Jin, Zhen Lian, Yuze Meng, Mark Blei, Takashi Taniguchi, Kenji Watanabe, Sefaattin Tongay, Wang Yao, Dmitry Smirnov, Chuanwei Zhang, Su-Fei Shi

**Affiliations:** 1 Department of Chemical and Biological Engineering, Rensselaer Polytechnic Institute, Troy, NY 12180 USA; 2 Department of Physics, The University of Texas at Dallas, Richardson, TX 75080 USA; 3 National High Magnetic Field Lab, Tallahassee, FL 32310 USA; 4 Department of Physics, Florida State University, Tallahassee, FL 32306 USA; 5 Kavli Institute, Cornell University, Ithaca, NY 14853 USA; 6 School for Engineering of Matter, Transport and Energy, Arizona State University, Tempe, AZ 85287 USA; 7 National Institute for Materials Science, 1-1 Namiki, Tsukuba, 305-0044 Japan; 8 Department of Physics, University of Hong Kong, Hong Kong, China; 9 Department of Electrical, Computer & Systems Engineering, Rensselaer Polytechnic Institute, Troy, NY 12180 USA

**Keywords:** Two-dimensional materials, Fluorescence spectroscopy

## Abstract

Strong many-body interaction in two-dimensional transitional metal dichalcogenides provides a unique platform to study the interplay between different quasiparticles, such as prominent phonon replica emission and modified valley-selection rules. A large out-of-plane magnetic field is expected to modify the exciton-phonon interactions by quantizing excitons into discrete Landau levels, which is largely unexplored. Here, we observe the Landau levels originating from phonon-exciton complexes and directly probe exciton-phonon interaction under a quantizing magnetic field. Phonon-exciton interaction lifts the inter-Landau-level transition selection rules for dark trions, manifested by a distinctively different Landau fan pattern compared to bright trions. This allows us to experimentally extract the effective mass of both holes and electrons. The onset of Landau quantization coincides with a significant increase of the valley-Zeeman shift, suggesting strong many-body effects on the phonon-exciton interaction. Our work demonstrates monolayer WSe_2_ as an intriguing playground to study phonon-exciton interactions and their interplay with charge, spin, and valley.

## Abstract (web summary)

An out-of-plane magnetic field is expected to strongly modify exciton-phonon interactions in atomically thin transitional metal dichalcogenides. Here, the authors show that the phonon-exciton interaction in monolayer WSe_2_ lifts the inter-Landau-level transition selection rules for dark trions.

## Introduction

Phonons, quasiparticles describing the collective vibrations of lattice, can strongly interact with excitons in semiconductor nanostructures^1–9^ and significantly influence the light emission and energy relaxation^2–4^. The strong Coulomb interaction and many-body effects in monolayer transition metal dichalcogenides (TMDCs)^10–13^ significantly enhance the phonon–exciton interaction, which was shown to reveal the silent Raman modes in boron nitride (BN) encapsulated monolayer WSe_2_^9,14^. The valley degree of freedom of the excitonic complexes also leads to types of phonon–exciton interactions that brighten the spin-forbidden dark exciton and intervalley exciton in WSe_2_ through chiral phonon modes at Γ point^5^ and K point^1^, respectively, resulting in phonon replica PL with long lifetime and large valley polarization. Due to the valley–spin locking^15,16^ and the nontrivial Berry phase^17–19^, the phonon–exciton interaction can be even more intriguing with the application of a large out-of-plane magnetic field, which induces valley-polarized Landau levels (LLs) and dictates the unique selection rules of the inter-LL transition^20–22^.

In this work, we explore the phonon–exciton interaction in the regime of Landau quantization. We achieve this through PL spectroscopy of a high-quality monolayer WSe_2_ device, which clearly resolves PL peaks from different phonon replicas, especially the phonon replica of the dark trions. We found that K phonons can lift the inter-LL transition selection rules of positive dark trions, resulting in the distinctively different Landau fan in the PL spectra compared with that from the bright trion. Exploiting the unique phonon–exciton interaction in WSe_2_, we can directly probe the hole LL in the valence band, bypassing the limitations of most optical spectroscopy techniques that can only probe the Landau quantization of combined electron–hole pairs. The simultaneous observation of the Landau quantization of the dark trion phonon replica and the bright trion also allows us to experimentally extract the electron and hole masses separately, which exhibit surprisingly large asymmetry, in stark contrast to common assumptions^23–25^. Interestingly, the onset of the phonon replica PL quantization also correlates with a drastic change of valley-Zeeman shift characterized by an increase of Landé g-factor from 5.5 to 18.0, suggesting strong many-body interactions.

## Results

### Phonon replicas of excitonic particles in monolayer WSe_2_

**Fig. 1:**
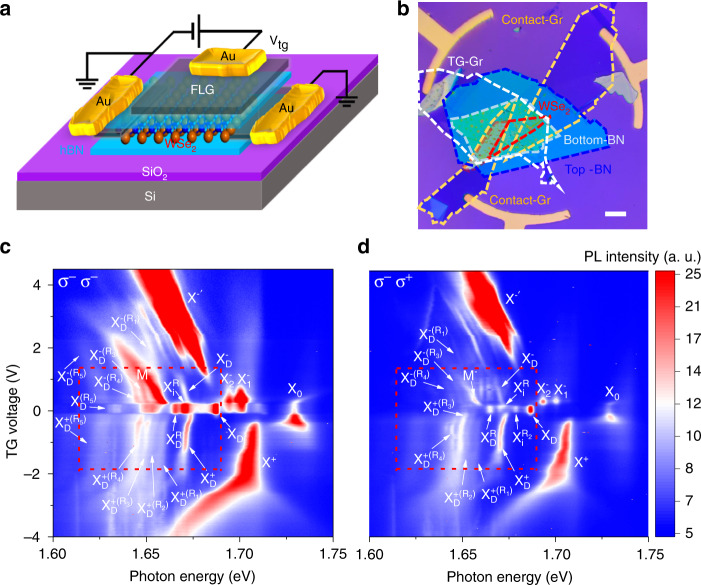
PL spectra of BN encapsulated monolayer WSe_2_ at 4.2 K. **a** Schematic of the h-BN encapsulated monolayer WSe_2_ device. **b** Optical microscope image of the device. The flakes of few-layer h-BN, few-layer graphene, and monolayer WSe_2_ are outlined with different colors. The scale bar is 20 µm. **c**, **d** PL spectra of the monolayer WSe_2_ as a function of top gate voltage for the σ^−^σ^−^ configuration (σ^−^ excitation and σ^−^ detection) and the σ^−^σ^+^ configuration (σ^−^ excitation and σ^+^ detection), respectively, with a CW laser excitation centered at 1.879 eV and an excitation power of 50 µW. The color represents the PL intensity. Besides the bright exciton (X_0_), dark exciton (X_D_), positive trion (X^+^), intervalley trion $({\mathrm{X}}_1^ - )$, intravalley trion $({\mathrm{X}}_2^ - )$, and the recently discovered positive dark trion $({\mathrm{X}}_{\mathrm{D}}^ + )$, negative dark trion $({\mathrm{X}}_{\mathrm{D}}^ - )$, intervalley exciton–phonon replica (${\mathrm{X}}_{\mathrm{i}}^{\mathrm{R}}$), and dark exciton–phonon replica $({\mathrm{X}}_{\mathrm{D}}^{\mathrm{R}})$, there are additional emerging excitonic states that are labeled as ${\mathrm{X}}_{\mathrm{D}}^{ + ({\mathrm{R}}_1)},{\mathrm{X}}_{\mathrm{D}}^{ - ({\mathrm{R}}_1)}$, ${\mathrm{X}}_{\mathrm{D}}^{ + ({\mathrm{R}}_2)}$, ${\mathrm{X}}_{\mathrm{D}}^{ + ({\mathrm{R}}_3)}$, ${\mathrm{X}}_{\mathrm{D}}^{ - ({\mathrm{R}}_3)}$, ${\mathrm{X}}_{\mathrm{D}}^{ + ({\mathrm{R}}_4)}$, ${\mathrm{X}}_{\mathrm{D}}^{ - ({\mathrm{R}}_4)}$, ${\mathrm{X}}_{\mathrm{D}}^{ + ({\mathrm{R}}_5)}$, and ${\mathrm{X}}_{\mathrm{D}}^{ - ({\mathrm{R}}_5)}$ in the electron- and hole-doping regions. In addition, ${\mathrm{X}}_{\mathrm{i}}^{{\mathrm{R}}_2}$ emerges in the σ^−^σ^+^ configuration.

The BN encapsulated monolayer WSe_2_ device was fabricated through a pickup method similar to previous reports^5,13,26^. The device structure is schematically shown in Fig. 1a, and a typical optical microscope image of the device is shown in Fig. 1b. We investigate the helicity-resolved PL spectra of the monolayer WSe_2_ as a function of the gate voltage at 4.2 K. The gate-voltage-dependent PL spectra in *σ*^−^*σ*^−^ (*σ*^−^ excitation and *σ*^−^ detection) and *σ*^−^*σ*^+^ (*σ*^−^ excitation and *σ*^+^ detection) configurations, shown in Fig. 1c, d, exhibit high spectral quality and a series of well-resolved PL peaks arising from various excitonic complexes. A significant number of the excitonic complexes have been identified previously, including the exciton (X_0_), intervalley n-trions (${\mathrm{X}}_1^ -$), intravalley n-trions (${\mathrm{X}}_2^ -$), dark exciton (X_D_)^27–29^, positive, and negative dark trions (${\mathrm{X}}_{\mathrm{D}}^ +$ and ${\mathrm{X}}_{\mathrm{D}}^ -$)^26,30,31^. Phonon replicas of the spin-forbidden dark exciton (${\mathrm{X}}_{\mathrm{D}}^{\mathrm{R}}$) and the momentum-dark intervalley exciton (${\mathrm{X}}_{\mathrm{i}}^{\mathrm{R}}$) have also been observed previously^1,5^, due to the long lifetime of the dark excitonic complexes. Because of the valley degree of freedom, angular momentum conservation has to be considered in the phonon–exciton interaction in TMDCs, and the chiral phonon mode is involved in ${\mathrm{X}}_{\mathrm{D}}^{\mathrm{R}}$ (linear combination of two doubly degenerate E″(Г) or ${\mathrm{X}}_{\mathrm{i}}^{\mathrm{R}}$ (LO(E′)(K))^1,5^. In the charge-neutral region (gate voltage from −0.10 to 0.21 V in Fig. 1c, d), one additional PL peak ${\mathrm{X}}_{\mathrm{i}}^{{\mathrm{R}}_2}$ (1.6788 eV in Fig. 1d) emerges in the *σ*^−^*σ*^+^ configuration due to its negative valley polarization. The ${\mathrm{X}}_{\mathrm{i}}^{{\mathrm{R}}_2}$ is another phonon replica of the intervalley exciton with the LA(K) phonon involved (see Supplementary Note 2 for details), consistent with a very recent report^32^.

**Fig. 2:**
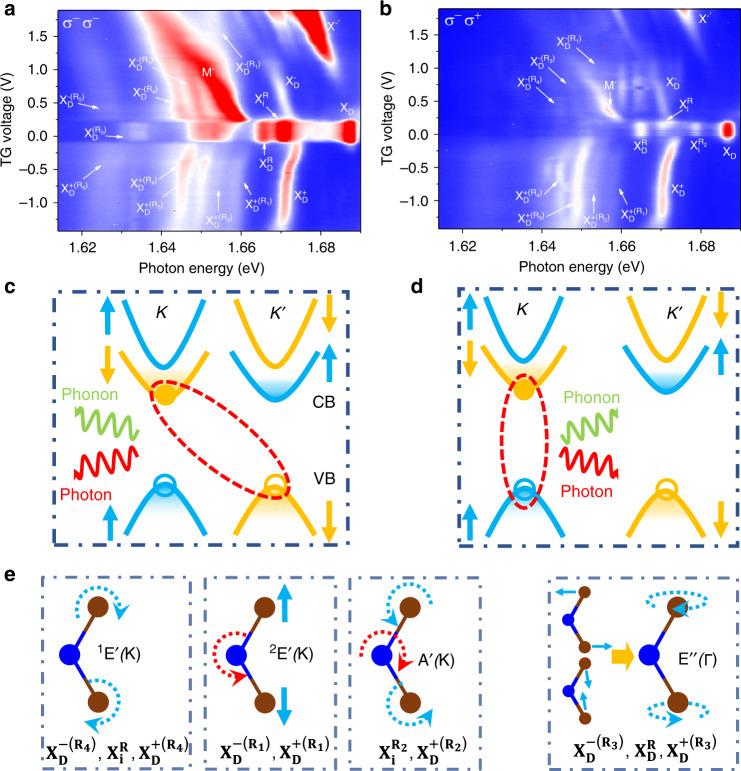
Phonon replicas of the dark trions. **a**, **b** Zoom-in color plots of the PL spectra for the boxed region (red dashed line) in Fig. 1c, d, respectively. The color represents the PL intensity. **c**, **d** Schematics of light emission from the positive dark trion state through the interlayer electron–hole recombination assisted by emitting a K phonon (**c**), or through the intralayer recombination assisted by emitting a Г phonon. **e** Illustration of different phonon modes and associated phonon replicas.

Interestingly, in the highly p-doped region, the PL of the bright trion peak (X^+^) exhibits a significant redshift after the gate voltage of approximately −2.5 V (Fig. 1a, b), and this gate dependence is closely followed by the dark trion (${\mathrm{X}}_{\mathrm{D}}^ +$), along with five PL peaks labeled as ${\mathrm{X}}_{\mathrm{D}}^{ + ({\mathrm{R}}_1)}$, ${\mathrm{X}}_{\mathrm{D}}^{ + ({\mathrm{R}}_2)}$, ${\mathrm{X}}_{\mathrm{D}}^{ + ({\mathrm{R}}_3)}$, ${\mathrm{X}}_{\mathrm{D}}^{ + ({\mathrm{R}}_4)}$, and ${\mathrm{X}}_{\mathrm{D}}^{ + ({\mathrm{R}}_5)}$ (Fig. 2a, b). This constant energy differences between each of the five emerging peaks and the dark trion for different gate voltages suggest that these five peaks are all phonon replicas (associated with different phonon modes) of the long-lived positive dark trion (or dark p-trion). Due to the three-particle nature of the positive dark trion, the electron–hole recombination can occur either in the same valley or across the opposite valley, assisted by **Γ** (Fig. 2d) or **K** phonons (Fig. 2c). The ${\mathrm{X}}_{\mathrm{D}}^{ + ({\mathrm{R}}_1)}$ and ${\mathrm{X}}_{\mathrm{D}}^{ + ({\mathrm{R}}_4)}$ peaks are lower than the dark trion peak by 13.1 and 26.4 meV, respectively, in good agreement with the theoretically calculated energy of the TA(K) phonon (11.6 to 11.7 meV)^1,2,32^ and LO(E′)(K) phonon (24.6 or 26.0 meV)^1,32^, respectively, (see Supplementary Table 1 for details). The phonon replica nature of the ${\mathrm{X}}_{\mathrm{D}}^{ + ({\mathrm{R}}_1)}$ and ${\mathrm{X}}_{\mathrm{D}}^{ + ({\mathrm{R}}_4)}$ peaks are also confirmed later by the measured Landé g-factor, which is the same as the positive dark trion within the experimental uncertainty. The ${\mathrm{X}}_{\mathrm{D}}^{ + ({\mathrm{R}}_2)}$ and ${\mathrm{X}}_{\mathrm{D}}^{ + ({\mathrm{R}}_3)}$ peaks are ~17.3 and ~21.6 meV below the positive dark trion ${\mathrm{X}}_{\mathrm{D}}^ +$, suggesting that they are the phonon replicas involving the LA(K) phonon (calculated energy of 16.8 to 17.0 meV)^1,2,32^ and E″(Г) (calculated energy of 21.8 meV)^5^, respectively, also consistent with a very recent report^32^. It is worth noting that the phonon energy can be experimentally extracted from the phonon replicas of the positive dark trion without worrying about the exchange interaction (see Supplementary Note 3 for details). As the phonon replicas associated with E″(Г), LO(E′)(K), and LA(K) modes are also found in the charge-neutral region, the corresponding peak of ${\mathrm{X}}_{\mathrm{D}}^{\mathrm{R}}$, ${\mathrm{X}}_{\mathrm{i}}^{\mathrm{R}}$, and ${\mathrm{X}}_{\mathrm{i}}^{{\mathrm{R}}_2}$ can then be used to determine the exchange interaction, which is extracted to be 9.3–10.1 meV (see Supplementary Note 3 for details). The phonon modes associated with ${\mathrm{X}}_{\mathrm{D}}^{ + ({\mathrm{R}}_1)}$, ${\mathrm{X}}_{\mathrm{D}}^{ + ({\mathrm{R}}_2)}$, ${\mathrm{X}}_{\mathrm{D}}^{ + ({\mathrm{R}}_3)}$, and ${\mathrm{X}}_{\mathrm{D}}^{ + ({\mathrm{R}}_4)}$ are schematically shown in Fig. 2e. The corresponding phonon mode replicas can also be found for the negative dark trion (or dark n-trion) except for ${\mathrm{X}}_{\mathrm{D}}^{ - ({\mathrm{R}}_2)}$, labeled as ${\mathrm{X}}_{\mathrm{D}}^{ - ({\mathrm{R}}_1)}$, ${\mathrm{X}}_{\mathrm{D}}^{ - ({\mathrm{R}}_3)}$, and ${\mathrm{X}}_{\mathrm{D}}^{ - ({\mathrm{R}}_4)}$ (Fig. 2a, b). ${\mathrm{X}}_{\mathrm{D}}^{ - ({\mathrm{R}}_2)}$ was not observed, possibly due to the strong PL at its neighborhood from the mysterious PL peak M^−^. The PL peaks of M^−^, ${\mathrm{X}}_{\mathrm{D}}^{ + ({\mathrm{R}}_5)}$, ${\mathrm{X}}_{\mathrm{D}}^{ - ({\mathrm{R}}_5)}$, and ${\mathrm{X}}_{\mathrm{D}}^{({\mathrm{R}}_5)}$ remain unidentified (see Supplementary Notes 5 and 7).

### Landau quantization in the PL spectra

**Fig. 3:**
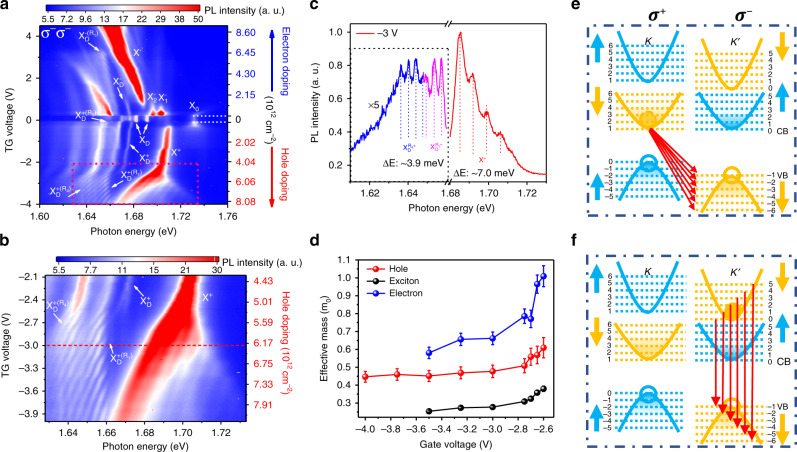
Magneto-PL spectra of BN encapsulated monolayer WSe_2_. **a** 2D color plot of PL spectra as a function of the top gate voltage. The X^+^, ${\mathrm{X}}_{\mathrm{D}}^{ + ({\mathrm{R}}_1)},{\mathrm{X}}_{\mathrm{D}}^{ - ({\mathrm{R}}_1)}$, and ${\mathrm{X}}_{\mathrm{D}}^{ + ({\mathrm{R}}_4)}$ peaks show clear Landau quantization. The color represents the PL intensity, and the calculated carrier density is shown in the right axis with region from −0.1 to 0.2 V being charge-neutral. **b** Zoom-in PL spectra for the boxed region in **a**. **c** The PL spectra for the gate voltage of −3 V (horizontal dashed line in **b**). The PL intensity in the energy range of ${\mathrm{X}}_{\mathrm{D}}^{ + ({\mathrm{R}}_1)}$ and ${\mathrm{X}}_{\mathrm{D}}^{ + ({\mathrm{R}}_4)}$ peaks (dashed box) is multiplied by five times. The oscillation spacing, ΔE, is ~3.9 meV for ${\mathrm{X}}_{\mathrm{D}}^{ + ({\mathrm{R}}_1)}$ and ${\mathrm{X}}_{\mathrm{D}}^{ + ({\mathrm{R}}_4)}$, and ~7.0 meV for X^+^. **d** The effective mass of hole, electron, and exciton in the hole-doping region. The error bar indicates the standard deviation of the LL spacing determined from the PL spectra. **e**, **f** Schematic of the inter-band LL transitions for different excitonic states: ${\mathrm{X}}_{\mathrm{D}}^{{\mathrm{R}}_1^ + },{\mathrm{X}}_{\mathrm{D}}^{{\mathrm{R}}_4^ + }$ (**e**) and X^+^ (**f**).

A large out-of-plane magnetic field not only lifts the valley degeneracy by lowering the valence band maximum of the K′ valley compared with that of the K valley (Fig. 3e, f); it also induces the valley-polarized LLs. We optically pump the WSe_2_ in the K′ valley and detect PL from the K′ valley (σ^−^σ^−^ configuration). In the highly p-doped WSe_2_ (boxed region in Fig. 3a, b), with an out-of-plane magnetic field of 17 T, we found Landau fan like oscillations emerging in the helicity-resolved PL spectra. These oscillations can be categorized into three sets: one set of parallel strips accompanying the positive bright trion (X^+^) and the other two sets accompanying the phonon replicas of the dark trion (${\mathrm{X}}_{\mathrm{D}}^{ + ({\mathrm{R}}_1)}$ and ${\mathrm{X}}_{\mathrm{D}}^{ + ({\mathrm{R}}_4)}$) (Fig. 3b). We attribute these oscillation features to the LL formation. The energy spacing between the oscillations increases linearly as a function of the magnetic field strength (see Supplementary Note 5), consistent with our interpretation.

The Landau fan patterns can be seen more clearly from PL spectra at the representative gate voltage of −3 V and B field of 17 T, shown in Fig. 3c (corresponding to the horizontal dashed line in Fig. 3b). The PL from the bright trion (X^+^) shows multiple peaks with even energy spacing of ~7.0 meV (red curve in Fig. 3c). The electron–hole recombination in the K′ valley is constrained by the valley-polarized selection rules in the K′ valley and only the n → −(n−1) transition is allowed^20,22^. At a given gate voltage, this gives rise to evenly space Landau fans with separation, which can be expressed phonematically as $\frac{{e\hbar B}}{{\mu ^ \ast }}$, where μ^*^ is the effective reduced mass of exciton. In addition, in the highly p-doped regime, the optically excited electron is mostly at the bottom of the conduction band. Therefore, PL intensity is strongest at the lowest energy peak (associated with the 0 → −1 inter-LL transition) and decreases quickly for higher-energy transition. These behaviors match well with our experimental observation in Fig. 3c. And we show the extracted value of μ^*^ as a function of the gate voltage in Fig. 3d.

Surprisingly, the Landau fan pattern from dark trion phonon replica ${\mathrm{X}}_{\mathrm{D}}^{ + ({\mathrm{R}}_1)}$ and ${\mathrm{X}}_{\mathrm{D}}^{ + ({\mathrm{R}}_4)}$ shows remarkably different behaviors: First, the energy spacing of the oscillations is ~3.9 ± 0.2 meV, about half of the value for the bright trion (~7.0 ± 0.2 meV). In addition, the intensity of the oscillations remains largely constant with energy for dark trion phonon replicas. These two distinctive differences suggest that the recombination processes underlying dark trion phonon replicas have very different inter-LL transition selection rules.

In a dark trion phonon replica, the recombination of the electron and hole can either occur in the same valley (K valley here) through emitting a **Γ** phonon or across the valley through emitting a **K** phonon (Fig. 2c). We do not observe the LL quantization associated with the **Γ** phonon replica of the positive dark trion, likely due to the weakened coupling with the **Γ** phonon in the presence of the large out-of-plane field (see Supplementary Note 4). For the **K** phonon replica, the electron can recombine with the hole in the other valley by emitting a **K** phonon. From the Fermi’s golden rule, the inter-LL transition rate from n in K valley to −n′ in K′ valley (minus sign means valence band) is given by^33^

1 $${\mathrm{P}}_{{\mathrm{n}} \to - {\mathrm{n}}^{\prime }} = \frac{{2\pi }}{\hbar }\mathop {\sum }\limits_\nu \left| {\mathop {\sum }\limits_m \frac{{\left\langle { - n^{\prime} , - } \right|H_{el}\left| {m, - } \right\rangle \left\langle {m, - } \right|H_{ep}\left| {n, + } \right\rangle }}{{E_{m, - } - E_{n, + } + \hbar {\upomega}_{{\mathbf{K}}{\upnu}}}}} \right|^2{\updelta}\left( {E_{n, + } - E_{ - n^{\prime} , - } - \hbar \omega - \hbar {\upomega}_{{\mathbf{K}}{\upnu}}} \right).$$

where *H*_*ep*_ is the electron−phonon interaction Hamiltonian and *H*_*el*_ is the electron-light coupling Hamiltonian (see Supplementary Note 10 for details). $\hbar {\upomega}_{{\mathbf{K}}{\upnu}}$ is the K phonon energy with mode *ν*. |n,+〉 (|n,−〉) represents the LL state in K (K′) valley. The selection rule for optical intra-LL transition is *n*′ = *m* + 1. Different from the optical process, the phonon scattering process $\langle m, - {\mathrm{|}}H_{ep}{\mathrm{|}}n, + \rangle$ is not limited by the selection rules (see Supplementary Note 10 for details). Thus, the phonon–exciton interaction involved here lifts the constraint of inter-LL transition selection rules (see Supplementary Note 10). As a result, electrons in the K valley can recombine with holes in any LLs in the K′ valley, as shown in Fig. 3d. The oscillation spacing will be determined solely by the $\Delta _{LL}^V$. In addition, the recombination probability will be similar for holes at different LLs in the valence band, as the occupation probability for holes in the first few LLs are all close to unity in the highly p-doped WSe_2_. This interpretation is consistent with the aforementioned experimental observations.

### Effective mass asymmetry between the electron and hole

Through the oscillation period of phonon replica of the positive dark trion, we can experimentally determine the LL spacing of the valence band $\Delta _{LL}^V = \frac{{e\hbar B}}{{m_h^ \ast }}$, where $m_h^ \ast$ is the effective hole mass and B is the magnetic field strength. For the gate voltage of −3 V, the extracted valence band LL spacing is ~3.9 ± 0.2 meV (Fig. 3c), corresponding to $m_h^ \ast$ ~ 0.50m_0_, with m_0_ being the free electron mass in the vacuum. Using the experimentally extracted oscillation spacing as a function of the gate voltage (see Supplementary Note 5), we can extract the gate-voltage-dependent effective hole mass as shown in Fig. 3d. The effective mass of the hole decreases from 0.61m_0_ to 0.45m_0_ as the voltage decreases from −2.6 to −4.0 V. The value of ~0.45m_0_ at the highest p-doping studied in this work (gate voltage of −4.0 V) is close to the theoretically predicted value of 0.4m_0,_ while the much-enhanced value of 0.61m_0_ indicates strong interactions at the gate voltage of −2.6 V.

At the same time, we also observe the LL quantization of the bright trion, which probes the combined Landau quantization of the exciton. Our top BN flake is ~9 nm thick, which corresponding to a gating efficiency of $2.15 \times 10^{12}\,{\rm{cm}}^{ - 2}\,{\rm{V}}^{ - 1}$ based on a geometry capacitance model (see Supplementary Note 9 for details). As a result, at the onset of the Landau fan of the bright trion at approximately −2.6 V, the density of hole is estimated to be $5.4 \times 10^{12}\,{\rm{cm}}^{ - 2}$ (onset of the p-doping at −0.1 V, Fig. 1). In this strongly p-doped regime, we expect a much reduced Coulomb interaction and assume a small exciton binding energy to help extract the effective electron mass^20^. For the loosely bound electron–hole pair^20^, the LL of the exciton $\frac{{e\hbar B}}{{\mu ^ \ast }}$ can be expressed as the summation of the LL spacing of the conduction band ($\Delta _{LL}^C$) and valence band $\Delta _{LL}^V$, i.e., $\Delta _{LL}^C + \Delta _{LL}^V = \frac{{e\hbar B}}{{\mu ^ \ast }}$_._ Therefore, we would have the expression $\frac{1}{{\mu ^ \ast }} = \frac{1}{{m_h^ \ast }} + \frac{1}{{m_e^ \ast }}$, which we can use to extract the effective electron mass of the electron, $m_e^ \ast$^19^. This picture is confirmed by the observation that the LL spacing from the exciton–phonon complex is always about half of the value for the bright trion for the gate voltage range we studied (Supplementary Fig. 11). Between the gate voltage −3.5 to −2.6 V, the extracted effective mass of the exciton varies from 0.25m_0_ to 0.38m_0_ (Fig. 3d), similar to what has been extracted from the absorption spectra of n-doped WSe_2_^20^. However, the associated effective electron mass is much larger than that of hole, 0.58m_0_ to 1.01m_0_ between the gate voltage −3.5 to −2.6 V. At the same experimental condition, this larger effective mass of electron than hole by as large as >50% (Fig. 3d), is in stark contrast to the typical assumption of electron–hole symmetry^20,23–25^.

The electron mass can also be directly extracted for n-doped WSe_2_, from the Landau quantization of the PL from the phonon replica of the negative dark trion (${\mathrm{X}}_{\mathrm{D}}^{ - ({\mathrm{R}}_1)}$, see Fig. 3a and Supplementary Note 5). The effective electron mass is extracted to be ~0.8m_0_ between the gate voltage of 2.25–2.85 V. This value falls in the range of the effective electron mass extracted from the p-doped WSe_2_ and is also significantly larger than the effective mass of hole.

### Gate-dependent valley-Zeeman shift

**Fig. 4:**
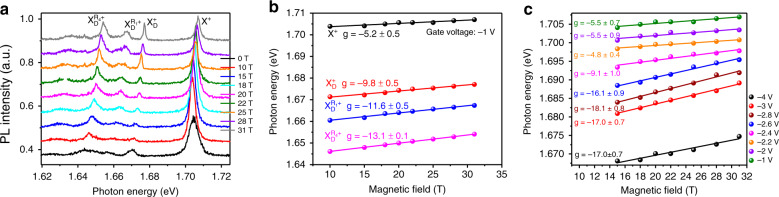
Valley-Zeeman shift from the magneto-PL spectra. **a** PL spectra in the σ^−^σ^−^ configuration at the gate voltage of −1 V for various magnetic field strengths. **b** The extracted PL peak position (solid dots) of X^+^, ${\mathrm{X}}_{\mathrm{D}}^ +$, ${\mathrm{X}}_{\mathrm{D}}^{ + ({\mathrm{R}}_1)},$ and ${\mathrm{X}}_{\mathrm{D}}^{ + ({\mathrm{R}}_4)}$ as a function of the magnetic field at 4.2 K for the gate voltage of −1 V. Solid lines are the linear fittings, with the extracted g-factor labeled. **c** The extracted g-factors of the bright positive trion X^+^ for different gate voltages.

Under the out-of-plane magnetic field, the PL peak of excitonic complexes from one particular valley will exhibit a linear shift due to the valley-Zeeman effect (Fig. 4a for K′ valley), which can be used to determine the g-factor and thus the nature of the excitonic complexes^1,5,13,26^. As shown in Fig. 4b, at the gate voltage of −1 V, the g-factor for the bright trion and dark positive trion is −5.2 and −9.8, respectively, consistent with the previous report^11,26,29,34^. For the dark trion phonon replica involving **K** phonons, the g-factor should be determined by the recombination of electron–hole pair from the opposite valleys, which is theoretically expected to be −12 (see Supplementary Note 6). This value agrees well with that of the dark trion replica, −11.6 for ${\mathrm{X}}_{\mathrm{D}}^{ + ({\mathrm{R}}_1)}$ and −13.1 for ${\mathrm{X}}_{\mathrm{D}}^{ + ({\mathrm{R}}_4)}$, confirming that these two PL peaks are the phonon replicas of the positive dark trion.

Interestingly, the magnitude of the g-factor of the bright trion increases significantly when the p-doping increases, with an abrupt change at the gate voltage of −2.6 V (Fig. 4c). This similar enhancement of the g-factor has been observed in the strongly n-doped WSe_2_ for the plasma mode^20,35^. This peculiarly abrupt enhancement of g-factor magnitude at the gate voltage of −2.6 V also closely correlates with the significant increase of the effective mass of electron and hole, suggesting the effects of many-body interactions. In fact, it has been shown that, in the highly p-doped regime, the simple trion picture should be better described with the picture of exciton–polaron^36^, in which the exciton interacts with all the holes in the opposite valley. Future exploration of the phonon–exciton interactions in the presence of valley-polarized LLs will strengthen our understanding of the exciting quantum many-body effects in the monolayer WSe_2_ platform.

## Discussion

In summary, we have shown the observation of Landau quantization of exciton–phonon complexes in monolayer WSe_2_ under a large out-of-plane magnetic field. Although optical spectroscopy has proven to be a powerful tool to study Landau quantization in two-dimension^37–39^, the two-particle nature makes it challenging to directly probe the electron or hole mass, and we cannot directly compare the obtained information from the optical spectroscopy with the single-particle information extracted from the low-temperature transport measurements. Here, taking advantage of the unique phonon–exciton interactions in WSe_2_, we bridge these two worlds through the sensitive PL spectroscopy, whose noncoherent nature helps reveal the single-particle information. The obtained electron mass and hole mass exhibit unexpected asymmetry, likely arising from many-body effects and will inspire future theoretical investigation. The advancement will be crucial for the understanding of mobility and thus the application of TMDCs-based devices. Further, the unique interaction between phonons and dark excitons in the presence of the valley-polarized LLs may shed light on potential avenues of manipulating spin and valley for valleytronics spintronics.

## Method

### Device fabrication

The BN encapsulated WSe_2_ device was fabricated through a pickup dry transfer method^5,13,26^. Monolayer WSe_2_, few-layer graphene, and few-layer BN were exfoliated onto 300 nm SiO_2_/Si substrate and identified with an optical microscope by the optical contrast. The few-layer BN flake, monolayer WSe_2_, two few-layer graphenes, and another few-layer BN flake were picked up by a PET stamp sequentially. The prepared stack was released onto the prepatterned Au electrodes by heating the stack up to 130 °C. The PET residue was removed by dissolving in chloroform for 2 h. Finally, another few-layer graphene was added onto the top BN to work as a top gate electrode, using the top BN as the dielectric.

### Magneto-PL measurements

The magneto-PL measurement was performed using a confocal micro-PL setup with the out-of-plane magnetic field. The circularly polarized excitation beam was set by a λ/4 waveplate from the linear polarized excitation laser and the laser was focused at the WSe_2_ sample by a ×50 objective (NA: ~0.65) to a spot size of ~2 μm. The collected PL was converted to the linear polarized light with the same λ/4 waveplate and analyzed by a CCD camera attached to a spectrograph. An assembly of a λ/2 waveplate and a second linear polarizer was used to distinguish between the (σ^+^σ^+^) and (σ^−^σ^−^) configurations.

## Supplementary information

SI

## Data Availability

The data that support the findings of this study are available from the authors on reasonable request, see author contributions for specific data sets.
