# Supplementary material for: Phonon-exciton Interactions in WSe2 under a quantizing magnetic field
Source: Nat Commun. 2020 Jun 19;11:3104. doi: 10.1038/s41467-020-16934-x (PMC7305315; doi:10.1038/s41467-020-16934-x)
Supplement: Supplementary file 1 — SI [file 41467_2020_16934_MOESM1_ESM.pdf]

Supplementary Information

Phonon-exciton Interactions in The Presence of Valley-polarized Landau Quantization

Zhipeng Li *et al.*

Supplementary Note 1. Intervalley and intravalley scattering through the conduction band

Here we discuss possible electron-hole pair recombination channels of dark excitonic complexes, focusing on the intervalley and intravalley scattering through the conduction band. To be consistent with the discussion in the main text, we assume that excitation light optically pumping the K' valley (σ^- excitation).

1) Charge-neutral region

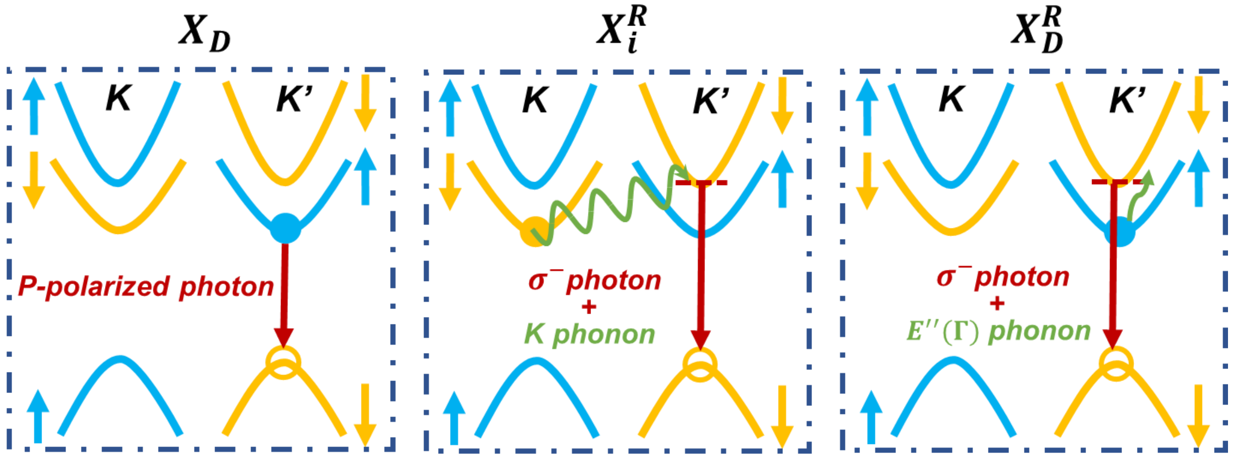

Supplementary Figure 1. Schematics of the dark exciton (X_D), **K** phonon replica (X_i^R) and **Γ** phonon replica (X_D^R) with their possible recombination paths.

The spin-forbidden dark exciton can still radiate through a finite out-of-dipole moment, not protected by the valley physics. As shown in Supplementary Fig. 1a, the radiated light is p-polarized, propagating in the plane of the WSe_2. The momentum-dark intervalley exciton can radiatively recombine by emitting a **K** phonon, and the electron can undergo an intervalley scattering to the upper conduction band of the opposite valley without flipping the spin, as shown in Supplementary Fig. 1b. This process corresponds to the X_i^R phonon replica peak reported in the main text, with the LO(E')(K) phonons involved[1,2]. The intravalley spin-forbidden dark exciton can also radiatively recombine by emitting a **Γ** phonon, and the electron can be scattered to the upper conduction band by flipping the spin. This corresponds to the X_D^R phonon replica in the main text, with the E" (Γ) phonon

involved. As we reported previously, the linear combination of the two degenerate linearly polarized E” (Γ) forms a chiral phonon mode to facilitate this intravalley scattering process to conserve the angular momentum[3].

2) Hole-doping region

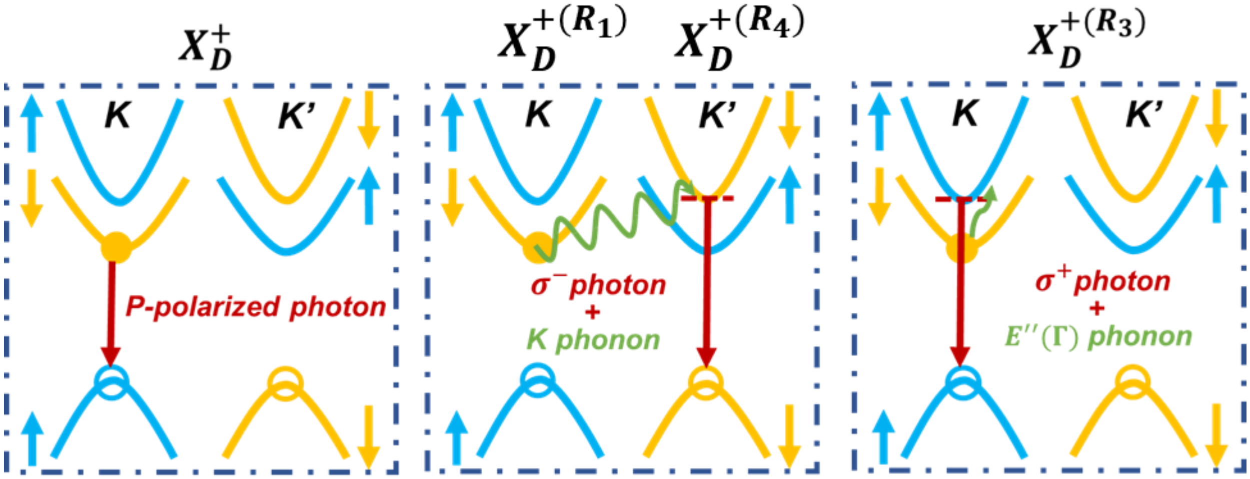

Supplementary Figure 2. Schematics of positive dark trion (X_D^+), its corresponding **K** phonon replica ($X_D^{+(R_4)}$ and $X_D^{+(R_1)}$) and **Γ** phonon replica ($X_D^{+(R_3)}$) with their possible recombination path.

In the p-doping region, we discuss the possible recombination channel for positive dark trions (dark p-trions). As we reported previously[4], the positive dark trion exhibits negative valley polarization under an out-of-plane magnetic field, suggesting that the favorable configuration after the K’ valley pumping should be as shown in Supplementary Fig. 2a. Due to the three-particle nature, the electron-hole pair recombination can occur either from the same valley or the opposite valley. As shown in Supplementary Fig. 2a, similar to the case of a charge-neutral spin-forbidden dark exciton, positive dark trion X_D^+ has a finite probability of emitting p-polarized light directly, without involving any phonons. The positive dark trion replicas can also emit light through a **K** phonon-assisted process (Supplementary Fig. 2b), with the electron at the K valley being scattered to the upper conduction band in the K’ valley. Therefore, the final light emission is through the bright exciton channel in K’ valley and the emitted photon will be of the helicity of σ^+. $X_D^{+(R_1)}$ and $X_D^{+(R_4)}$ phonon replica peak reported in the main text, with the TA(K) and LO(E’)(K) phonons involved[1,2]. The positive dark trion can also radiatively recombine through the electron-hole pair in the same valley (K), assisted by a chiral **Γ** phonon that mixes bright and dark exciton channel[3], emitting a σ^+ photon through the bright exciton channel. This process corresponds to the $X_D^{+(R_3)}$ discussed in the main text, which involves E” (Γ) phonon.

3) Electron-doping region

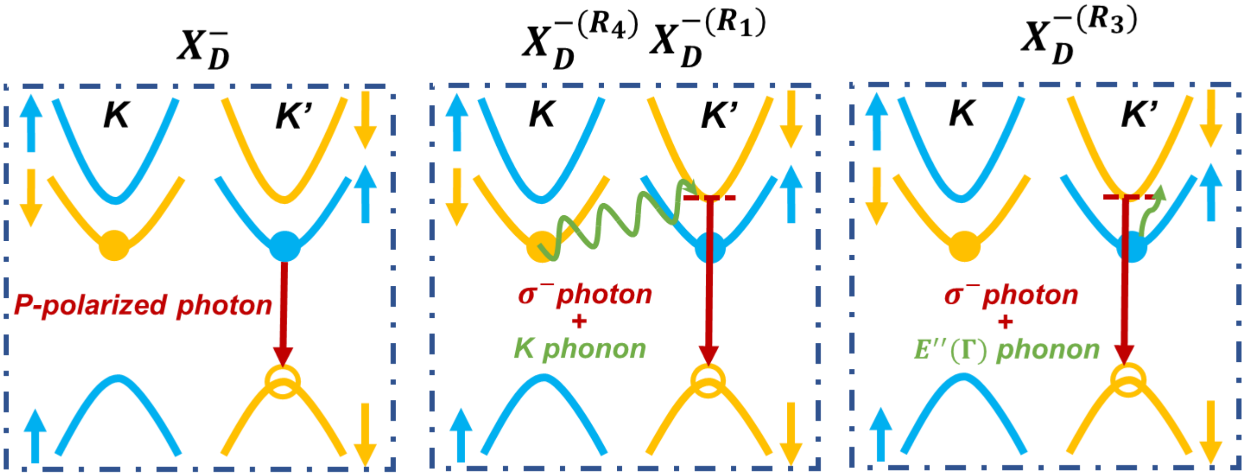

Supplementary Figure 3. Schematics of negative dark trion (X_D^-), its corresponding **K** phonon replica ($X_D^{-(R_1)}$ and $X_D^{-(R_4)}$) and **Γ** phonon replica ($X_D^{-(R_3)}$) with their possible recombination path.

As it is more difficult for the hole to be scattered to the opposite valley, the more favored configuration of the negative dark trion (dark n-trion) is shown in Supplementary Fig. 3a, which could emit p-polarized phonon through the electron and hole in the same valley, similar to the dark exciton and positive dark trion. The negative dark trion could also recombine with the assistance of a **K** phonon, by scattering the electron in the K valley to the up-conduction band in the K' valley without flipping the spin. The consequent emission through the bright exciton channel in K' valley results in a σ^- photon. $X_D^{-(R_1)}$ and $X_D^{-(R_4)}$ peaks discussed in the main text correspond to this process, which involves the TA(K) and LO(E')(K) phonons[1,2], respectively. Again, due to the three-particle nature, the negative dark trion can also radiatively recombine through the electron-hole in the same (K' here) valley, which can be assisted with the chiral **Γ** phonon, emitting a σ^- photon through the bright exciton channel. $X_D^{-(R_3)}$ discussed in the main text corresponds to this process, with the E" (Γ) phonon involved.

Supplementary Note 2. Intervalley scattering through valence band

In this session, we also consider the second valance band in K and K' valley, which are typical ~500 meV away from the first valence band in the WSe_2[5]. Potentially, the intervalley exciton, shown in Supplementary Fig. 4a, can radiatively recombine by scattering the hole in the K' valley to the second valence band in K valley, with the spin conserved. This process can also be realized through emitting a **K** phonon. The resulted hole in the K valley (second valence band) and recombined with the electron in the same valley and emit a σ^+ photon. Due to the large energy difference between the two valence

bands, the possibility of intervalley scattering through the valence band is low and the associated phonon replica PL is of less intensity than the phonon replica only involving the intervalley scattering through the valence band. The reported LA(K) phonon replica of the intervalley exciton phonon replica ($X_i^{R_2}$) is from such a process. $X_i^{R_2}$ shows negative valley polarization compared to other excitonic complexes, consistent with this interpretation and a recent report[1].

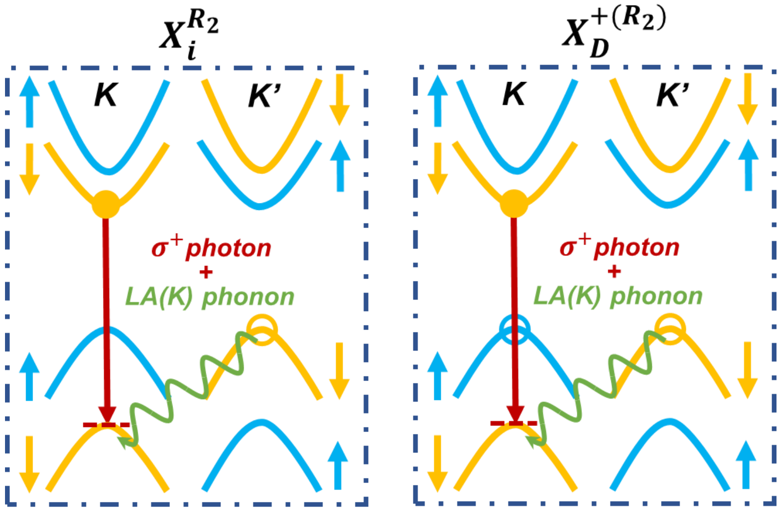

Supplementary Figure 4. Schematics of dark exciton replica ($X_i^{R_2}$) and negative dark trion replica ($X_i^{+(R_2)}$) with their possible recombination path through the valence band.

Similarly, such a process can also be found in the radiative recombination of the positive dark trion, as shown in Supplementary Fig. 4b. The reported $X_D^{+(R_2)}$ in the main text is from such a process involving the same phonon mode, LA(K) phonon replica of the dark trion. It is also less intense than the other K phonon replicas of the positive dark trions and vanishes under the application of an out-of-plane magnetic field. That explains the absence of the Landau quantization effect on $X_D^{+(R_2)}$.

Supplementary Note 3. Exchange interaction

The energy difference of intravalley exciton and intervalley exciton originates from the exchange interaction, shown in Supplementary Fig. 5. The clearly resolved phonon replica PL offers a way to determine this exchange interaction experimentally. For example, the energy of the intervalley exciton, shown in Supplementary Fig. 5b, can be determined by the energy difference between the peak position of dark exciton (X_D) and the intervalley exciton phonon replica (such as X_i^R or $X_i^{R_2}$), associated phonon modes energy, and the exchange interaction. The phonon mode energy can be experimentally determined from the energy difference between the positive dark phonon and its replica, without worrying about the exchange interaction. Therefore, we determine the exchange

interaction to be 9.3-10.1 meV, depending on which mode we use to extract the exchange interaction. The detailed results are shown in Supplementary Table 1.

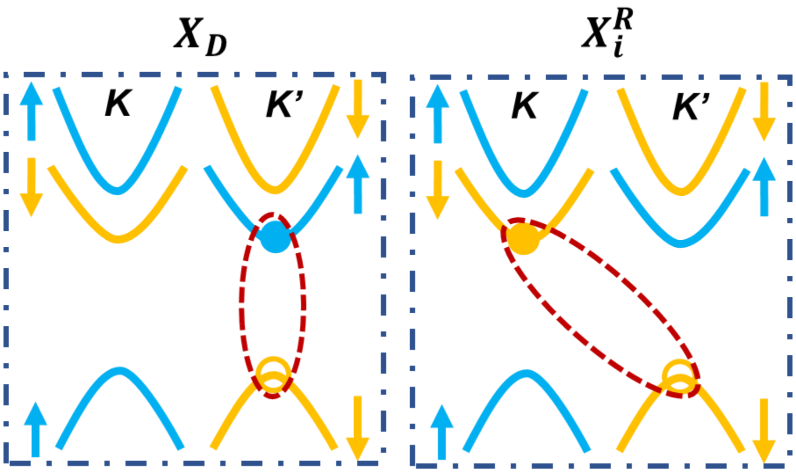

Supplementary Figure 5. Schematics of dark exciton ($\mathrm{X_D}$) and **K** phonon replica ($\mathrm{X_i^R}$).

Supplementary Table 1. Energy of replica and determined phonon mode.[1–3,7]

	Energy of replica peak (eV)		Energy of parents peak (eV)		Energy shift (meV)	Phonon energy (meV)	Exchange interaction (meV)	Phonon mode	Theoretical energy (meV)
Neutral region ($\mathrm{V_g = 0\ V}$)	$\mathrm{X_D^R}$	1.6656	$\mathrm{X_D}$	1.6872	21.6	21.6	/	$E''(\Gamma)$	21.8 [ref 3]
	$\mathrm{X_i^R}$	1.6706	$\mathrm{X_D}$	1.6872	16.6	26.4 (Hole-doping)	9.8	$LO(E')(K)$	24.6 [ref 2,7], 26.0 [ref 1]
						26.7 (Electron-doping)	10.1		
	$\mathrm{X_i^{R_2}}$	1.6788	$\mathrm{X_D(\sigma^-\sigma^+)}$	1.6868	8.0	17.3	9.3	$LA(K)$	16.8 [ref 2,7], 17.0 [ref 1]
	$\mathrm{X_D^{R_5}}$	1.6343	$\mathrm{X_D}$	1.6872	52.9	52.9	/		
Electron doping region ($\mathrm{V_g = 0.4\ V}$)	$\mathrm{X_D^{-(R_1)}}$		$\mathrm{X_D^-}$	1.6713			/		
	$\mathrm{X_D^{-(R_3)}}$	1.6497	$\mathrm{X_D^-}$	1.6713	21.6	21.6	/	$E''(\Gamma)$	21.8 [ref 3]
	$\mathrm{X_D^{-(R_4)}}$	1.6446	$\mathrm{X_D^-}$	1.6713	26.7	26.7	/	$LO(E')(K)$	24.6 [ref 2,7], 26.0 [ref 1]
	$\mathrm{X_D^{-(R_5)}}$	1.6265	$\mathrm{X_D^-}$	1.6713	44.8	44.8	/		
Hole doping region ($\mathrm{V_g = -0.4\ V}$)	$\mathrm{X_D^{+(R_1)}}$	1.6594	$\mathrm{X_D^+}$	1.6725	13.1	13.1	/	$TA(K)$	11.6 [ref 7], 11.7 [ref 2]
	$\mathrm{X_D^{+(R_2)}}$	1.6552	$\mathrm{X_D^+}$	1.6725	17.3	17.3	/	$LA(K)$	16.8 [ref 2,7], 17.0 [ref 1]
	$\mathrm{X_D^{+(R_3)}}$	1.6509	$\mathrm{X_D^+}$	1.6725	21.6	21.6	/	$E''(\Gamma)$	21.8 [ref 3]
	$\mathrm{X_D^{+(R_4)}}$	1.6461	$\mathrm{X_D^+}$	1.6725	26.4	26.4	/	$LO(E')(K)$	24.6 [ref 2,7], 26.0 [ref 1]
	$\mathrm{X_D^{+(R_5)}}$	1.6268	$\mathrm{X_D^+}$	1.6725	45.7	45.7	/		

With the understanding of the possible recombination pathways in mind, the phonon modes associated with the phonon replica can be analyzed accordingly. As discussed in the main text, the phonon modes can be determined through energy conservation, momentum conservation, valley polarization and g-factor compared with the parent PL peak.[1–3,6,7]. The energy of dark trion and trion replicas can be extracted from the PL spectra, and they are summarized in Supplementary Table 1, along with the analyzed phonon mode energy. For the phonon replica involving the exchange interaction, the

phonon energy was experimentally determined from the dark trion replica, which was then used to determine the exchange interaction.

Supplementary Note 4. PL intensity of Γ phonon replica of dark exciton under an out-of-plane magnetic field

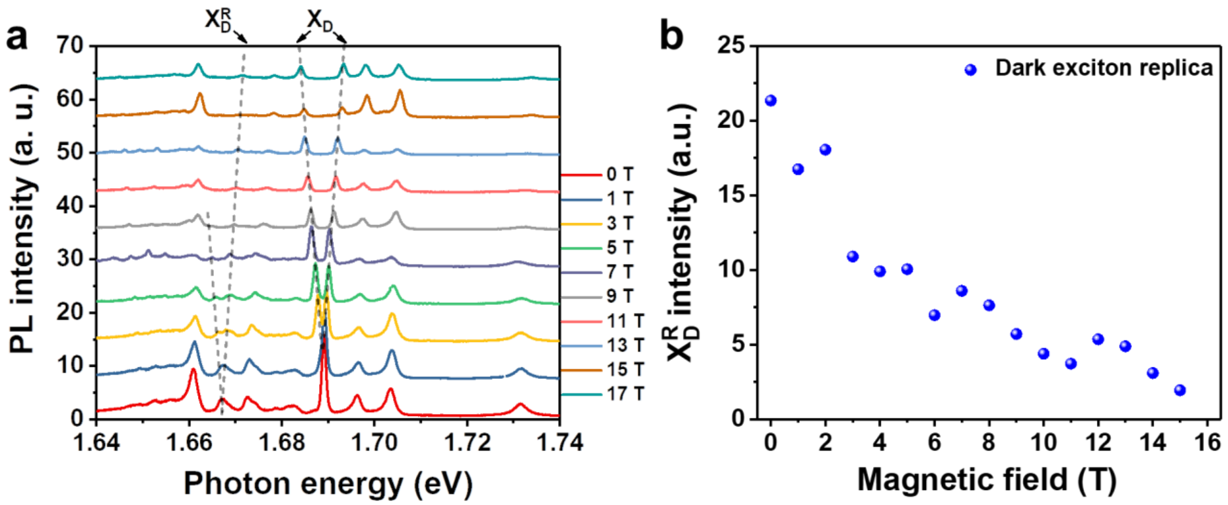

Supplementary Figure 6. PL intensity of dark exciton (X_D) and its **Γ** phonon replica (X_D^R) under the out-of-plane magnetic field. (a) PL spectra of intrinsic WSe_2 for various magnetic field strengths. (b) PL intensity of **Γ** phonon replica of the dark exciton as a function of the magnetic field.

The PL intensity of the phonon replica (X_D^R) is one indication of the coupling strength of the electron with $E''(\Gamma)$ phonon. As the magnetic field strength increases, the intensity of **Γ** phonon replica of the dark exciton (X_D^R) decreases, suggesting a decreasing scattering possibility of electron assisted by the $E''(\Gamma)$ phonon. Those observations support our explanation that, under a high magnetic field, the $E''(\Gamma)$ phonon replica of the positive dark trion can barely be observed.

Supplementary Note 5. Magneto-PL spectra and discussion of the effective mass.

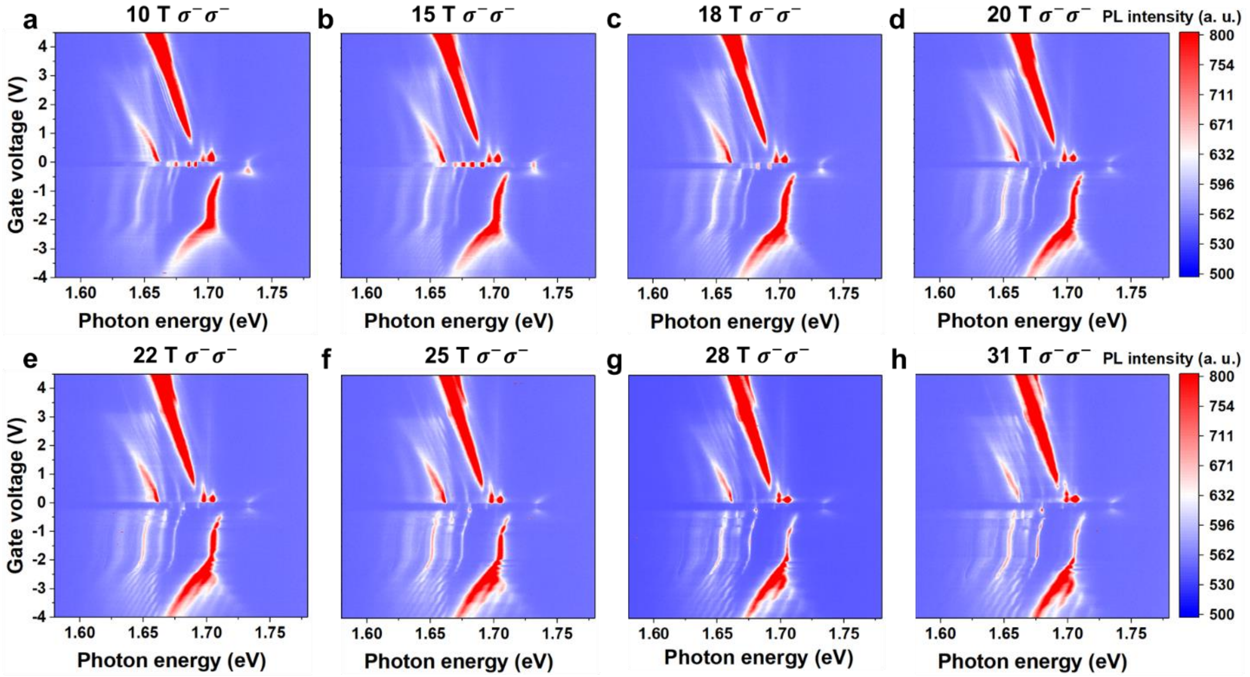

Supplementary Figure 7. Color plot of PL spectra as a function of gate voltage under different magnetic fields. The color represents the PL intensity.

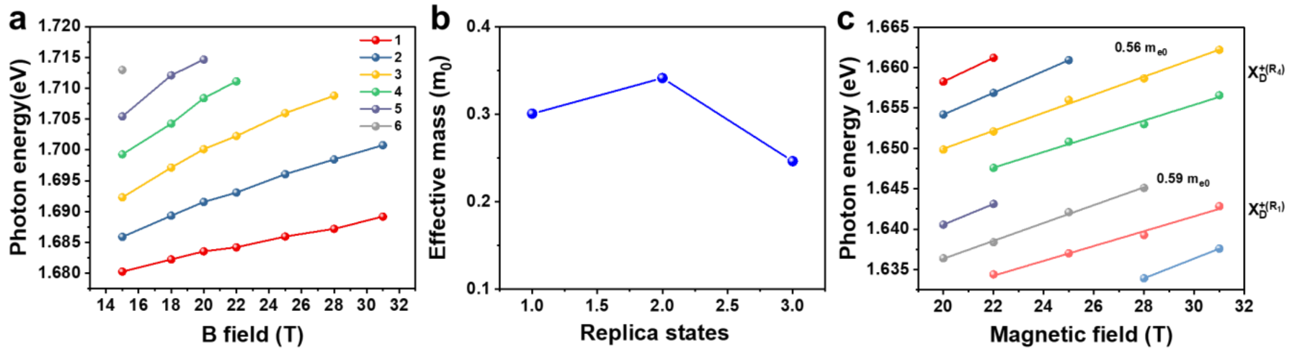

Supplementary Figure 8. Effective mass extracted from the energy spacing of the positive trion and positive dark trion replica states. (a) Photon energy of bright positive trion peak (X^+) under different magnetic fields. (b) Exciton effective mass for different Landau levels of positive trion. (c) Positive dark trion replica peaks ($X_D^{+(R_1)}$ and $X_D^{+(R_4)}$) at the gate voltage of -3 V under different magnetic fields. The extracted effective mass of hole is 0.56 m_0 and 0.59 m_0 for $X_D^{+(R_4)}$ and $X_D^{+(R_1)}$, respectively.

We extract the bright positive trion peak (X^+) and positive dark trion peaks ($X_D^{+(R_1)}$ and $X_D^{+(R_4)}$) at the gate voltage of -3 V under different magnetic fields. Then we calculated the

exciton effective mass from the positive trion peak, as well as the effective hole mass from the positive dark trion peaks.

The linear dependence of trion and trion replica with the magnetic field is evidence of the existence of the Landau level splitting of the conduction band and the valence band.

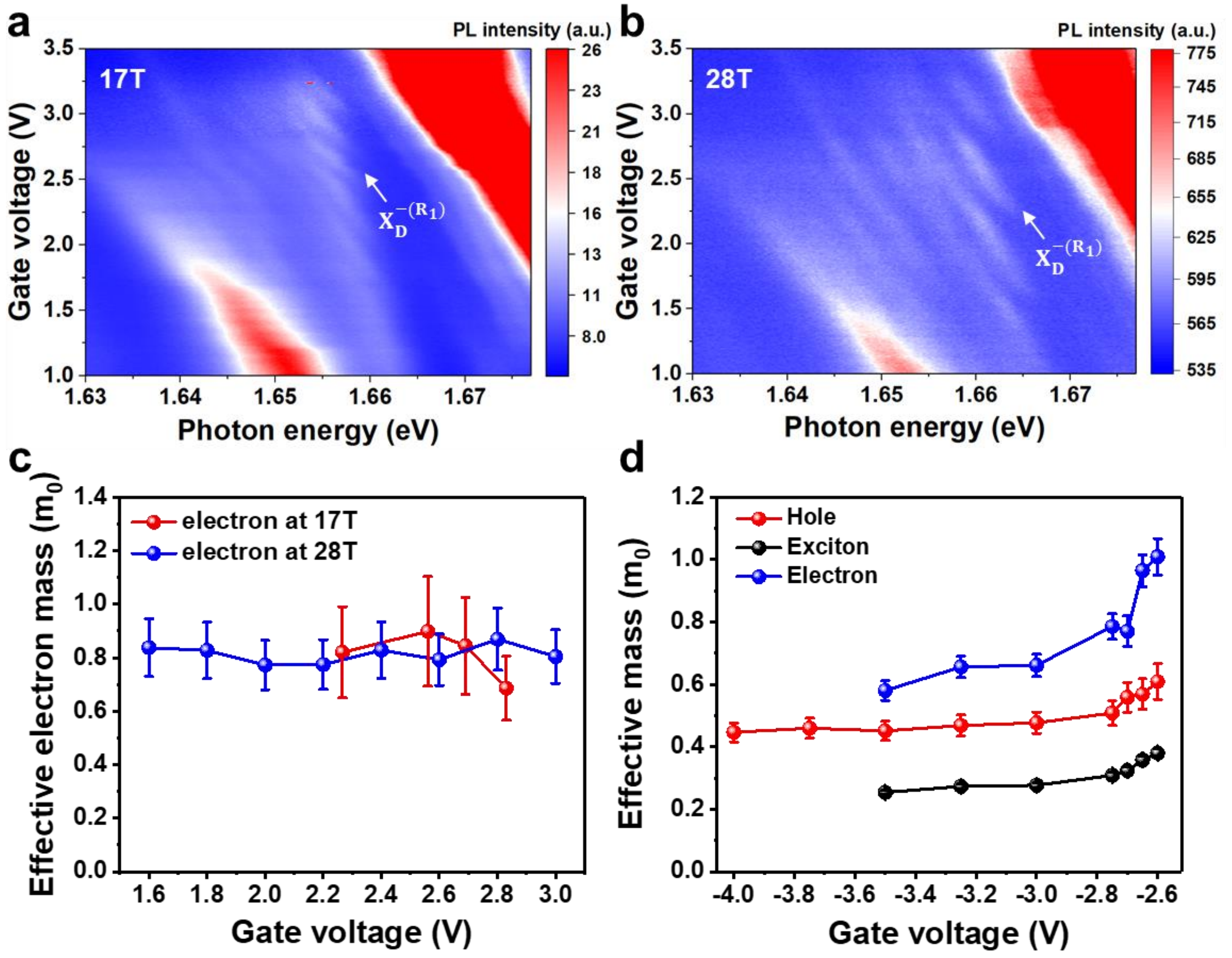

Supplementary Figure 9. (a, b) Zoom-in color plot of PL spectra as a function of the top gate voltage at 17 T (a) and 28 T (b), respectively. The color represents the PL intensity. $X_D^{-(R_1)}$ clearly shows Landau quantization. (c) The effective mass of electron in the electron-doping region. (d) The effective mass of hole, electron and exciton in the hole-doping region for comparison. This panel is shown in the main text as Fig. 3d. The error bar indicates the standard deviation of the LL spacing determined from the PL spectra.

Supplementary Note 6. g-factor of intervalley exciton

Based on the tight-binding model[8–10], orbital contribution g_O, valley contribution g_{valley}, and spin contribution g_S are the three components in the general formula for the g-factor

of each band, which is systematically deduced in the previous reports[2,3,11]. Here we include the calculated results of different excitonic states in Supplementary Table 2.

Supplementary Table 2. The calculated *g*-factors for the exciton, dark exciton and trion-exciton complex.

Peaks	Spectral *g*-factor
**Bright exciton (X_0)**	-4.0
**Positive trion (X^+)**	-4.0
**Dark exciton (X_D)**	-8.0
**Intervalley exciton (X_i)**	-12.0

As the g-factor will be only determined by the electron-hole pair involved in the radiative recombination, the theoretically expected value of the **K** phonon replica of the positive dark trion will be the same as the intervalley exciton, -12. The **Γ** phonon replica of the positive trion will be the same as the dark exciton, -8.0.

Supplementary Note 7. Line traces for different excitonic states

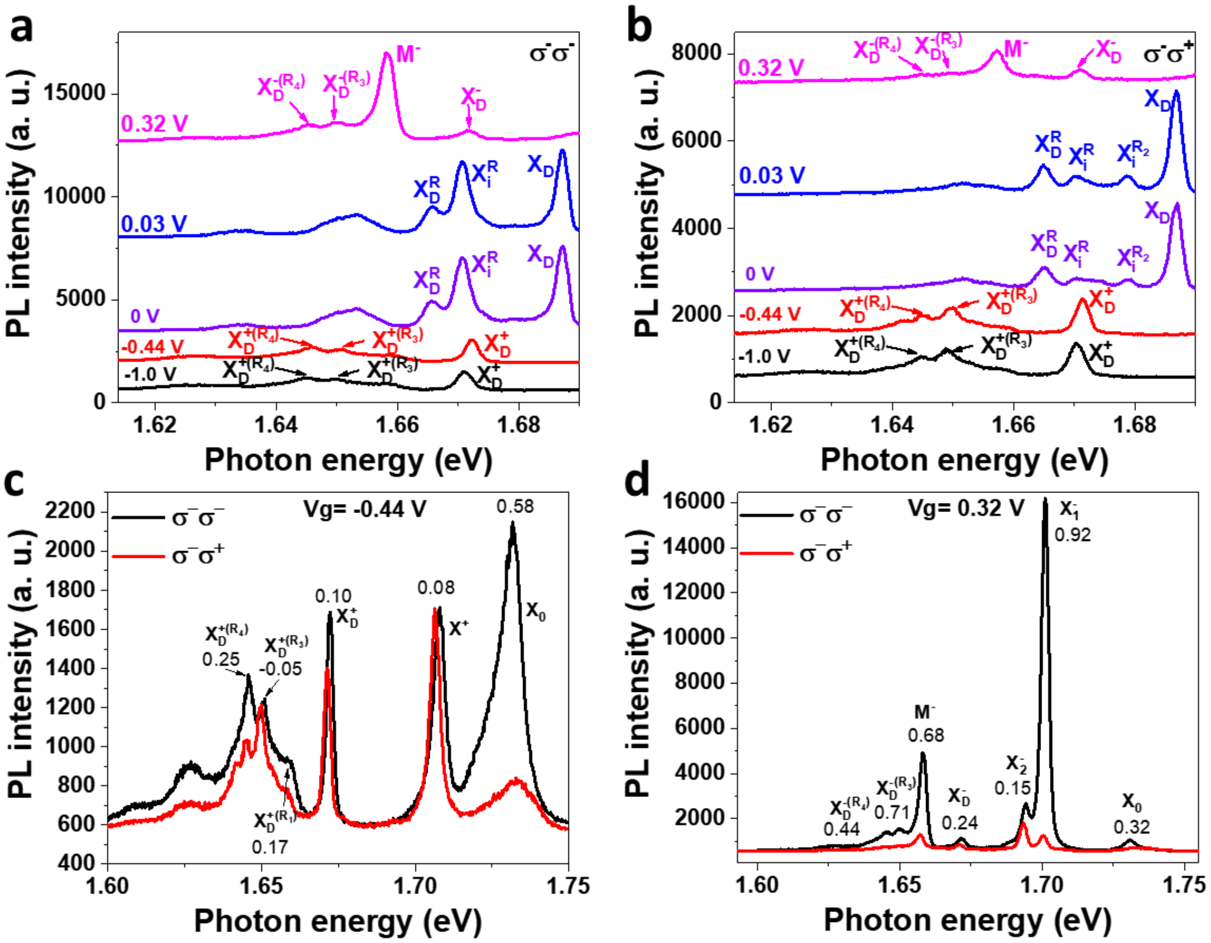

Supplementary Figure 10. Line traces for different excitonic states. (a,b) Line cuts for the configurations of $\sigma^- \sigma^-$ (a) and $\sigma^- \sigma^+$ (b) at different gate voltages corresponding to Fig 2a and 2b in the main text. (c,d) The extracted valley polarization for different excitonic states at different gate voltages of -0.44 V (c) and 0.32 V (d), corresponding to the positive and negative charged regions, respectively.

Supplementary Note 8. LL spacing for the exciton-phonon complex and bright trion in the p-doping regime.

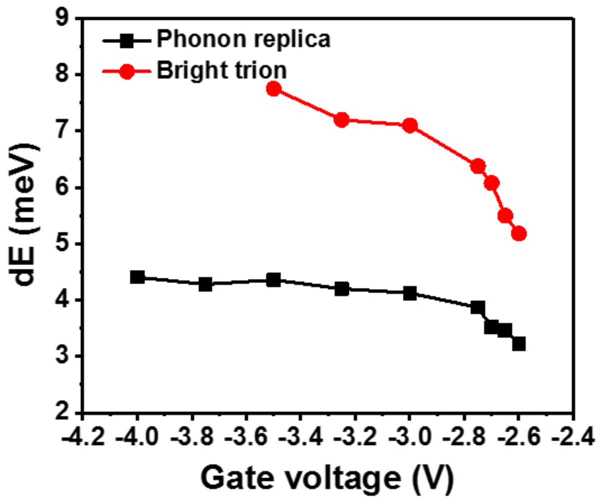

Supplementary Figure 11. LL spacing extracted for in the p-doping region for different gate voltages.

Supplementary Note 9. Calculation of doping density

We use a simple parallel capacitance model to determine the doping density. The effective capacitance per unit area of the device is given by $C = \frac{\varepsilon_0 \varepsilon_{BN}}{t}$, where ε_0 is the vacuum permittivity and ε_{BN} is the dielectric constant of h-BN, estimated as 3.5 in our calculation[12,13]. t is the thickness of the h-BN, which is ~ 9 nm for our device. The doping efficiency is thus calculated to be $2.15 \times 10^{12}\ cm^{-2}\ V^{-1}$.

Using the capacitance model above, the doping density is calculated to be $5.4 \times 10^{12}\ cm^{-2}$ at the gate voltage of -2.6 V (with the onsets of p-doping at -0.1 V and n-doping at 0.2 V, respectively, see Fig. 1 in the main text), which is comparable to the high doping at the turning point $7 \times 10^{12}\ cm^{-2}$ (complete filling of the first LL)[14]. At the gate voltage of -4.0 V, the hole doping density is $\sim 8.4 \times 10^{12}\ cm^{-2}$.

Supplementary Note 10. Theoretical calculation

For the phonon replica of dark trion, the combination of electron and hole could occur either in the same valley through a $\boldsymbol{\Gamma}$ phonon or across the valley through a K phonon. Here we discuss the second process.

1. The electron-phonon coupling

The atomic displacement can be expressed in terms of ladder operator of phonon as[12],

$$\Delta\tau_{\kappa\alpha p} = \sqrt{\frac{M_0}{N_p M_\kappa}} \sum_{\boldsymbol{q}\nu} e^{i\boldsymbol{q}\cdot\boldsymbol{R_p}}\, e_{\kappa\alpha,\nu}(\boldsymbol{q}) l_{\boldsymbol{q}\nu}\left(\hat{b}_{\boldsymbol{q}\nu} + \hat{b}^{\dagger}_{-\boldsymbol{q}\nu}\right). \tag{1}$$

Here M_0 is the proton mass, N_p is the number of unit cell and R_p is the coordinates of unit cell *p*, τ denote the nucleus type and M_κ is the corresponding mass. $l_{\boldsymbol{q}\nu} = \sqrt{\hbar/(2M_0\omega_{\mathbf{q}\nu})}$, $\omega_{\mathbf{q}\nu}$ and $e_{\kappa\alpha,\nu}$ denote the phonon frequency and polarization, respectively.

The lattice vibration (phonon) changes potential $V(\boldsymbol{r};\{\tau_{\kappa\alpha p}\})$ applied on electrons. The potential to the first order in displacement is given by

$$V(\mathbf{r};\{\tau_{\kappa\alpha p}\}) = V(\mathbf{r};\{\tau^0_{\kappa\alpha p}\}) + \sum_{\kappa\alpha p}\frac{\partial V}{\partial\tau_{\kappa\alpha p}}\Delta\tau_{\kappa\alpha p}. \tag{2}$$

With Supplementary Equation 1, we have

$$V(\mathbf{r};\{\tau_{\kappa\alpha p}\}) = V(\mathbf{r};\{\tau^0_{\kappa\alpha p}\}) + \sqrt{\frac{1}{N_p}}\sum_{\boldsymbol{q}\nu}\Delta_{\boldsymbol{q}\nu} V\left(\hat{b}_{\boldsymbol{q}\nu} + \hat{b}^{\dagger}_{-\boldsymbol{q}\nu}\right). \tag{3}$$

$$\Delta_{\boldsymbol{q}\nu} V = e^{i\mathrm{q}\cdot\mathrm{r}}\Delta_{\boldsymbol{q}\nu} v, \quad \Delta_{\boldsymbol{q}\nu} v = l_{\boldsymbol{q}\nu}\sum_{\kappa\alpha}\sqrt{\frac{M_0}{M_\kappa}}\, e_{\kappa\alpha,\nu}(\boldsymbol{q})\partial_{\kappa\alpha,\boldsymbol{q}} v. \tag{4}$$

$$\partial_{\kappa\alpha,\boldsymbol{q}} v = \sum_p e^{-i\boldsymbol{q}\cdot(\boldsymbol{r}-\boldsymbol{R}_p)}\frac{\partial V}{\partial\tau_{\kappa\alpha p}}\Big|_{\boldsymbol{r}-\boldsymbol{R}_p}. \tag{5}$$

We can see that $\Delta_{\boldsymbol{q}\nu} v$ and $\partial_{\kappa\alpha,\boldsymbol{q}} v$ are lattice-periodic functions. The electron-phonon interaction Hamilton can be expressed as

$$H_{ep} = \sum_{n\mathbf{k},n'\mathbf{k}'}\langle\psi_{n\mathbf{k}}|V(\{\tau_{\kappa\alpha p}\}) - V(\{\tau^0_{\kappa\alpha p}\})|\psi_{n'\mathbf{k}'}\rangle\, \hat{a}^{\dagger}_{n\mathbf{k}}\hat{a}_{n'\mathbf{k}'}. \tag{6}$$

With the expressions above, we have

$$H_{ep} = \sqrt{\frac{1}{N_p}}\sum_{\mathrm{k},\mathbf{q},mn\nu} g_{mn\nu}(\mathrm{k},\mathrm{q})\hat{a}^{\dagger}_{m,\mathbf{k}+\mathbf{q}}\hat{a}_{n\mathrm{k}}\left(\hat{b}_{\boldsymbol{q}\nu} + \hat{b}^{\dagger}_{-\boldsymbol{q}\nu}\right). \tag{7}$$

The coupling matrix $g_{mn\nu}(\mathbf{k},\mathbf{q}) = \langle u_{m,\mathbf{k+q}}|\Delta_{q\nu} v|u_{n,\mathbf{k}}\rangle_{\mathrm{uc}}$, where $|u_{n,k}\rangle$ is the periodic part of Bloch wavefunction and the integral is over the unit cell. The electron-phonon coupling matrix $g_{mn\nu}$ is different from the optical dipole transition matrix. There is not selection rule during the electron-phonon scattering process.

2. Phonon-assisted optical emission under magnetic field

Now we discuss the recombination process of dark trion through phonon in K valley under the magnetic field. Near K and K' valley, the bands form Landau levels (LLs). The electron-phonon interaction in Supplementary Equation 7 can be rewritten in the LL basis and only **K** (**K'**) valley phonon participates conserving the momentum. The phonon-assisted optical emission is shown in Supplementary Fig.12.

For the phonon-assisted optical transition, the total Hamiltonian is given by

$$\mathrm{H_{tot}} = H_e + H_{ph} + H_{ep} + H_{eL}. \tag{8}$$

H_e is the electron single-particle Hamiltonian, H_{ph} is the phonon Hamiltonian, H_{ep} the electron-phonon coupling Hamiltonian and H_{el} is the electron-light interaction Hamiltonian, with the expressions given as follows

$$H_e = \sum_{nl\tau s} E_{n\tau s}\hat{a}^\dagger_{nl\tau s}\hat{a}_{nl\tau s}. \tag{9}$$

$$\mathrm{H_{ph}} = \sum_{\nu,\pm}\hbar\omega_{\pm\mathbf{K}\nu}\left(\hat{b}^\dagger_{\pm\mathbf{K}\nu}\hat{b}_{\pm\mathbf{K}\nu}+\frac{1}{2}\right). \tag{10}$$

$$H_{ep} = \sqrt{\frac{1}{N_p}}\sum_{n'l's',nls,\nu} g^\nu_{n'l's',nls}(\mathbf{K})\hat{a}^\dagger_{n'l',\tau=-,s'}\hat{a}_{nl,\tau=+,s}\left(\hat{b}_{\mathbf{K}\nu}+\hat{b}^\dagger_{-\mathbf{K}\nu}\right). \tag{11}$$

$$H_{eL} = \sum_{n'l'nl,\tau,s}\hat{a}^\dagger_{n'l'\tau s}\hat{a}_{nl\tau s}d_{n'l',nl,\tau s}. \tag{12}$$

$E_{n\tau s}$ is the LL energy with n, l denoting LL quantum number[13] ($l = -|n|, -|n|+1, \cdots$ is the angular momentum of LL states), $\tau = \pm$ and s denoting the valley and spin, respectively. $d_{n'l',nl,\tau s}$ is the optical dipole transition matrix. The electron-phonon coupling matrix is given by

$$g^\nu_{n'l's',nls}(\mathbf{K}) = \langle n'l',\tau=-,s'|\Delta_{\mathbf{K}\nu}v|nl,\tau=+,s\rangle. \tag{13}$$

The integral is over the entire 2D quantized region, and $|nl\tau s\rangle$ is the LL state, which in spatial space is the LL envelope function multiplied by the band-edge wavefunction (periodic part of Bloch wavefunction),

$$\langle\boldsymbol{r}|nl\tau s\rangle = \psi_{nl\tau s}(\boldsymbol{r})u_\tau(\boldsymbol{r}). \tag{14}$$

The LL envelope function has the form[13]

$$\psi_{nl\tau s}(\boldsymbol{r}) = e^{il\theta}f_{nl\tau s}(|\boldsymbol{r}|). \tag{15}$$

where θ is the spatial angle. Due to the C_3 symmetry, band-edge wavefunction has the form

$$u_\tau(\boldsymbol{r}) = e^{il_\tau\theta}\sum_{j_\tau} e^{3ij_\tau\theta}u_{j_\tau}(|\boldsymbol{r}|),\ (j_\tau \text{ is an integer}). \tag{16}$$

where l_τ is the valley pseudoangular momentum (PAM). For the conduction band, $l_+ = 1$ in K valley and $l_- = -1$ in K' valley. Also for the phonon part, $\Delta_{\mathbf{K}\nu}v$ has a similar form

$\Delta_{\mathbf{K}\nu} v(\boldsymbol{r}) = e^{il_{ph}\theta} \sum_{j_{ph}} e^{3ij_{ph}\theta} u_{j_{ph}}(|\boldsymbol{r}|)$ (j_{ph} is integer), l_{ph} is the phonon PAM[14]. So the coupling matrix in Supplementary Equation 13 will give the relation $l' - l = 3(j_{ph} + j_+ - j_-) + l_+ - l_- + l_{ph}$. For simplicity, we can define $J = 3(j_{ph} + j_+ - j_-) + l_+ - l_- + l_{ph}$. A certain value of J contains all the combinations of j_{ph}, j_+ and j_- with the relation given above. The radial integral usually does not vanish. There is no explicit constraint between n' and n in Supplementary Equation 13.

The phonon-assisted optical transition rate from **K** valley to **K**' valley can be obtained in second-order perturbation theory[15]. Here we only consider the $\sigma-$ polarized light emission. From the Fermi's golden rule, the transition rate is given by

$$\mathrm{P}_{\mathrm{n}\to -\mathrm{n}'} = \frac{2\pi}{\hbar} \sum_{\nu} \left| \sum_{m,ll'l''} \frac{\langle n'l', -|H_{el}|ml'', -\rangle\langle ml'', -|H_{ep}|nl, +\rangle}{E_{m,-} - E_{n,+} + \hbar\omega_{\mathbf{K}\nu}} \right|^2 \delta\left(E_{n,+} - E_{-n',-} - \hbar\omega - \hbar\omega_{\mathbf{K}\nu}\right) = \frac{2\pi}{\hbar N_p} \sum_{\nu} \left| \sum_{m,ll'l''} \frac{d_{n'l',ml'',-}\, g^{\nu}_{ml'',nl,}}{E_{m,-} - E_{n,+} + \hbar\omega_{\mathbf{K}\nu}} \right|^2 \delta\left(E_{n,+} - E_{-n',-} - \hbar\omega - \hbar\omega_{\mathbf{K}\nu}\right). \quad (17)$$

Here we neglect the spin index as during the whole process the spin is conserved. The phonon-assisted optical transition process is illustrated in Supplementary Fig.12.

For the optical transition matrix part, there is a selection rule, i.e. $n'' \to -(n'' + 1)$ in K' valley for left-polarized light[15]. So we have $n' = m + 1,\ l' = l'' = l + J$. No constraints between m LL and initial LL n. Then the transition rate can be reformulated as

$$\mathrm{P}_{\mathrm{n}\to -\mathrm{n}'} = \frac{2\pi}{\hbar N_p} \sum_{\nu} \left| \sum_{l,J} \frac{d_{n',(l+J),(n'-1),(l+J),-}\, g^{\nu}_{(n'-1),(l+J),nl,}}{E_{m,-} - E_{n,+} + \hbar\omega_{\mathbf{K}\nu}} \right|^2 \delta\left(E_{n,+} - E_{-n',-} - \hbar\omega - \hbar\omega_{\mathbf{K}\nu}\right). \quad (18)$$

From Supplementary Equation 18 and Supplementary Fig.12, we can see the transition from $n = 1$ LL in K valley to -1 LL in K' valley is through a K phonon to be scattered to $n' = 0$ LL state in K' valley and then to fall to -1 LL by emitting a $\sigma-$ polarized photon. Also, the transitions from $n = 1$ LL in K valley to -2 and other LLs are allowed, thanks to the lacking of selection rule during electron-phonon scattering process.

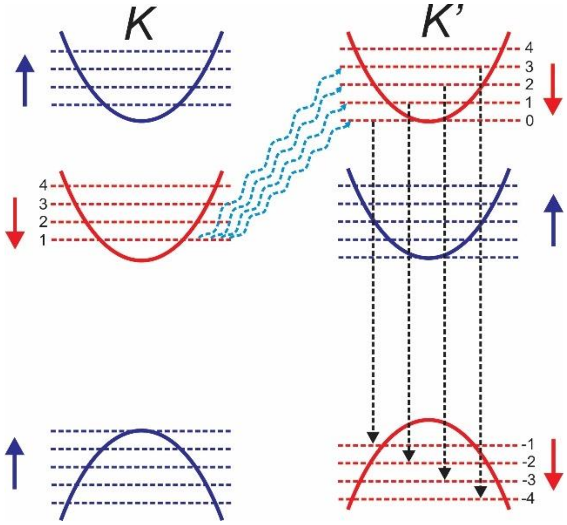

Supplementary Figure 12. Illustration of phonon-assisted optical transition. The black dashed wavy line denotes the phonon process while the black dashed straight line is the optical process. We neglect the Zeeman shift of the band edge.

Supplementary References

1. He, M. *et al.* Valley Phonons and Exciton Complexes in a Monolayer Semiconductor. *Nat. Commun.* **11**, 618 (2020).

2. Li, Z. *et al.* Momentum-Dark Intervalley Exciton in Monolayer Tungsten Diselenide Brightened via Chiral Phonon. *ACS Nano* **13**, 14107–14113 (2019).

3. Li, Z. *et al.* Emerging photoluminescence from the dark-exciton phonon replica in monolayer WSe_2. *Nat. Commun.* **10**, 2469 (2019).

4. Li, Z. *et al.* Direct Observation of Gate-Tunable Dark Trions in Monolayer WSe_2. *Nano Lett.* **19**, 6886–6893 (2019).

5. Xiao, D., Liu, G.-B., Feng, W., Xu, X. & Yao, W. Coupled spin and valley physics in monolayers of MoS_2 and other group-VI dichalcogenides. *Phys. Rev. Lett.* **108**, 196802 (2012).

6. Liu, E., Baren, J. Van, Lu, Z., Taniguchi, T. & Watanabe, K. Valley-selective chiral phonon replicas of dark excitons and trions in monolayer WSe_2. *Phys. Rev. Res.* **1**, 032007 (2019).

7. Zhu, H. *et al.* Observation of chiral phonons. *Science.* **359**, 579–582 (2018).

8. Macneill, D. *et al.* Breaking of valley degeneracy by magnetic field in monolayer $MoSe_2$. *Phys. Rev. Lett.* **114**, 037401 (2015).

9. Srivastava, A. *et al.* Valley Zeeman effect in elementary optical excitations of

monolayer WSe_2. *Nat. Phys.* **11**, 141–147 (2015).

10. Aivazian, G. *et al.* Magnetic control of valley pseudospin in monolayer WSe_2. *Nat. Phys.* **11**, 148–152 (2015).

11. Li, Z. *et al.* Revealing the Biexciton and Trion-exciton Complexes in BN Encapsulated WSe_2. *Nat. Commun.* **9**, 3719 (2018).

12. Laturia, A., Van de Put, M. L. & Vandenberghe, W. G. Dielectric properties of hexagonal boron nitride and transition metal dichalcogenides: from monolayer to bulk. *npj 2D Mater. Appl.* **2**, 6 (2018).

13. Kim, K. K. *et al.* Synthesis and Characterization of Hexagonal Boron Nitride Film as a Dielectric Layer for Graphene Devices. *ACS Nano* **6**, 8583–8590 (2012).

14. Wang, Z., Shan, J. & Mak, K. F. Valley- and spin-polarized Landau levels in monolayer WSe_2. *Nat. Nanotechnol.* **12**, 144–149 (2017).

15. Giustino, F. Electron-phonon interactions from first principles. *Rev. Mod. Phys.* **89**, 015003 (2017).
